# Sintilimab-induced toxic epidermal necrolysis complicated in advanced gastric cancer: a case report and literature review

**DOI:** 10.3389/fimmu.2026.1771194

**Published:** 2026-03-02

**Authors:** Qi Zhao, Rui-Ke Cao, Hai-Peng Zou, Rui-Bin Wang, Ling-Xia Yu, Wei Zhang, Li-Na Wang, Yan-Dong Miao

**Affiliations:** 1 Cancer Center, Yantai Affiliated Hospital of Binzhou Medical University, The Medical College of Binzhou Medical University, Yantai, China; 2 Hainan Academy of Medical Sciences, Hainan Medical University, Haikou, China; 3 Department of Medical Aesthetics, Burns and Plastic Surgery, Yantai Affiliated Hospital of Binzhou Medical University, The Medical College of Binzhou Medical University, Yantai, China; 4 Department of Pharmacy, Yantai Affiliated Hospital of Binzhou Medical University, The Medical College of Binzhou Medical University, Yantai, China; 5 Guangdong Provincial Key Laboratory of Medical Biomechanics, National Key Discipline of Human Anatomy, School of Basic Medical Sciences, Southern Medical University, Guangzhou, China; 6 Department of Oncology, Xinhui District People’s Hospital, Jiangmen, China

**Keywords:** gastric cancer, immune checkpoint inhibitor, immune-related adverse event, multidisciplinary management, PD-1 inhibitor, sintilimab, Stevens–Johnson syndrome, toxic epidermal necrolysis

## Abstract

**Background:**

Immune checkpoint inhibitors (ICIs) can trigger immune-related adverse events (irAEs), among which Stevens–Johnson syndrome/toxic epidermal necrolysis (SJS/TEN) is exceptionally rare but potentially fatal. Sintilimab, a PD-1 inhibitor increasingly used for advanced gastric cancer, has only sporadically been linked to SJS/TEN.

**Case presentation:**

We report a 60-year-old man with metastatic gastric adenocarcinoma who developed TEN 7 days after the first sintilimab infusion. He presented with rapidly progressive diffuse erythema, bullae, and epidermal detachment involving >90% of the body surface area, accompanied by fever and upper gastrointestinal bleeding leading to hemodynamic instability.

**Interventions:**

Sintilimab was permanently discontinued. Multidisciplinary management was initiated, including intensive fluid and electrolyte resuscitation, high-dose intravenous immunoglobulin, systemic corticosteroids with careful bleeding surveillance, specialized burn-type wound care, tailored anti-infective therapy based on susceptibility testing, and aggressive nutritional support. This case report was prepared in accordance with the CARE (CAse REport) guidelines.

**Outcomes:**

Skin lesions gradually re-epithelialized, infection was controlled, and gastrointestinal bleeding stabilized. The patient fully recovered from TEN and was discharged on hospital day 32. No recurrence or secondary infection was observed on follow-up.

**Conclusion:**

Although effective for gastric cancer, sintilimab may rarely induce life-threatening TEN. Early recognition, immediate ICI withdrawal, and coordinated multidisciplinary care are pivotal to survival. This case highlights the importance of early recognition, immediate discontinuation of ICIs, and coordinated multidisciplinary management in patients who develop life-threatening cutaneous immune-related adverse events.

## 1 Introduction

Immune checkpoint inhibitors (ICIs), particularly programmed death-1/ligand 1 (PD-1/PD-L1) blockade, have reshaped systemic therapy for multiple malignancies, including advanced gastric cancer. However, by unleashing antitumor immunity, ICIs may also provoke immune-related adverse events (irAEs) (1). Although the overall incidence of fatal irAEs is low (approximately 0.3–1.3% of treated patients) (1, 2), their diverse manifestations and high mortality demand vigilant monitoring as immunotherapy use expands. Among these, severe dermatologic toxicities like Stevens–Johnson syndrome (SJS) and toxic epidermal necrolysis (TEN) – sometimes termed bullous epidermal necrolysis (BEN) in drug-induced cases – are particularly ominous. TEN carries an acute mortality of around 30% (3).

In the field of advanced gastric cancer, PD-1 inhibitors have become an important treatment modality, improving survival in a subset of patients. The CheckMate-649 trial demonstrated that adding nivolumab to chemotherapy significantly prolonged overall survival in first-line therapy for gastric and gastroesophageal cancers (4). Likewise, the ORIENT-16 trial conducted in China showed encouraging efficacy of sintilimab plus chemotherapy, leading to its widespread clinical use (5).

With the increasing adoption of ICIs in gastroenterologic oncology, clinicians are encountering a broad spectrum of irAEs. Most immune toxicities are mild, such as transient rash or endocrinopathies; however, 10–20% of patients receiving PD-1 monotherapy develop high-grade irAEs (1). Dermatologic irAEs are among the most frequent; maculopapular rash and pruritus are reported in up to ~20–30% of patients on PD-1 inhibitors, usually low-grade (6). In contrast, SJS/TEN represents an exceedingly rare irAE, characterized by widespread blistering, epidermal detachment, and mucosal involvement – essentially a catastrophic failure of the skin barrier.

Pharmacovigilance analyses estimate that PD-1/PD-L1 inhibitor–induced SJS/TEN occurs at a rate of roughly 6 per 10,000 patients (0.06%), about two to three times higher than that seen with classical high-risk drugs such as allopurinol or carbamazepine (6). Notably, PD-1-related SJS/TEN tends to have a delayed onset (median ≈16 weeks after treatment initiation, compared with 4–8 weeks for conventional drugs), suggesting a distinct immunopathogenic mechanism (6).

Pathologically, SJS and TEN are regarded as part of a spectrum of bullous epidermal necrolysis (BEN), differing mainly by the extent of skin loss (TEN involving > 30% of body surface area) (7). These reactions are typically driven by T-cell-mediated cytotoxicity against keratinocytes. In the context of ICIs, PD-1 blockade may unleash autoreactive CD8^+^ T cells that target skin epithelia or heighten immune reactivity to concomitant drug antigens (6). Clinically, PD-1 induced SJS/TEN often presents with prodromal fever and diffuse rash involving not only the skin but also ocular, oral, and genital mucosa – a pattern mirroring classic SJS/TEN but possibly with multisystem immune activation.

Importantly, PD-1 inhibitor–associated SJS/TEN (BEN) is so rare that existing evidence is derived almost entirely from isolated case reports and small case series. A recent review identified only 28 published cases of sintilimab-related cutaneous adverse reactions, with SJS/TEN reported sporadically in patients with thymic carcinoma, lung cancer, melanoma, and other malignancies (8, 9). In advanced gastric cancer, where ICIs have only recently entered standard practice, published experience with such severe skin irAEs remains exceptionally sparse (10).

This knowledge gap presents a major clinical challenge: no standardized guidelines or large-scale studies currently exist to direct the management of PD-1 inhibitor–induced SJS/TEN, particularly in the complex setting of gastric cancer where tumor-related bleeding, malnutrition, and concurrent cytotoxic therapy are common. Consequently, clinicians must extrapolate from general SJS/TEN management principles and small immunotherapy case reports, highlighting an urgent need for systematic research and evidence-based treatment recommendations (11).

We report here a case of a metastatic gastric cancer patient who developed bullous epidermal necrolysis (SJS/TEN) as an irAE after treatment with sintilimab, complicated by concurrent gastrointestinal bleeding. The goal of this case study is to detail the full clinical course from onset to recovery, and to derive lessons for early recognition and management of such catastrophic toxicity. We hypothesize that prompt identification, immediate withdrawal of the PD-1 inhibitor, and aggressive multidisciplinary intervention were pivotal in controlling the reaction and achieving a favorable outcome. Through this report, we aim to illustrate how coordination among specialists – including dermatology (for diagnosis and skin-directed therapy), oncology (for cancer control and immunotherapy cessation), critical care/burn unit teams (for wound care and hemodynamic support), gastroenterology or surgery (for hemorrhage management), and nutrition support – can together surmount a life-threatening irAE. This case report was prepared in accordance with the CARE (CAse REport) guidelines (12).

## 2 Patient information

**Figure 1:**
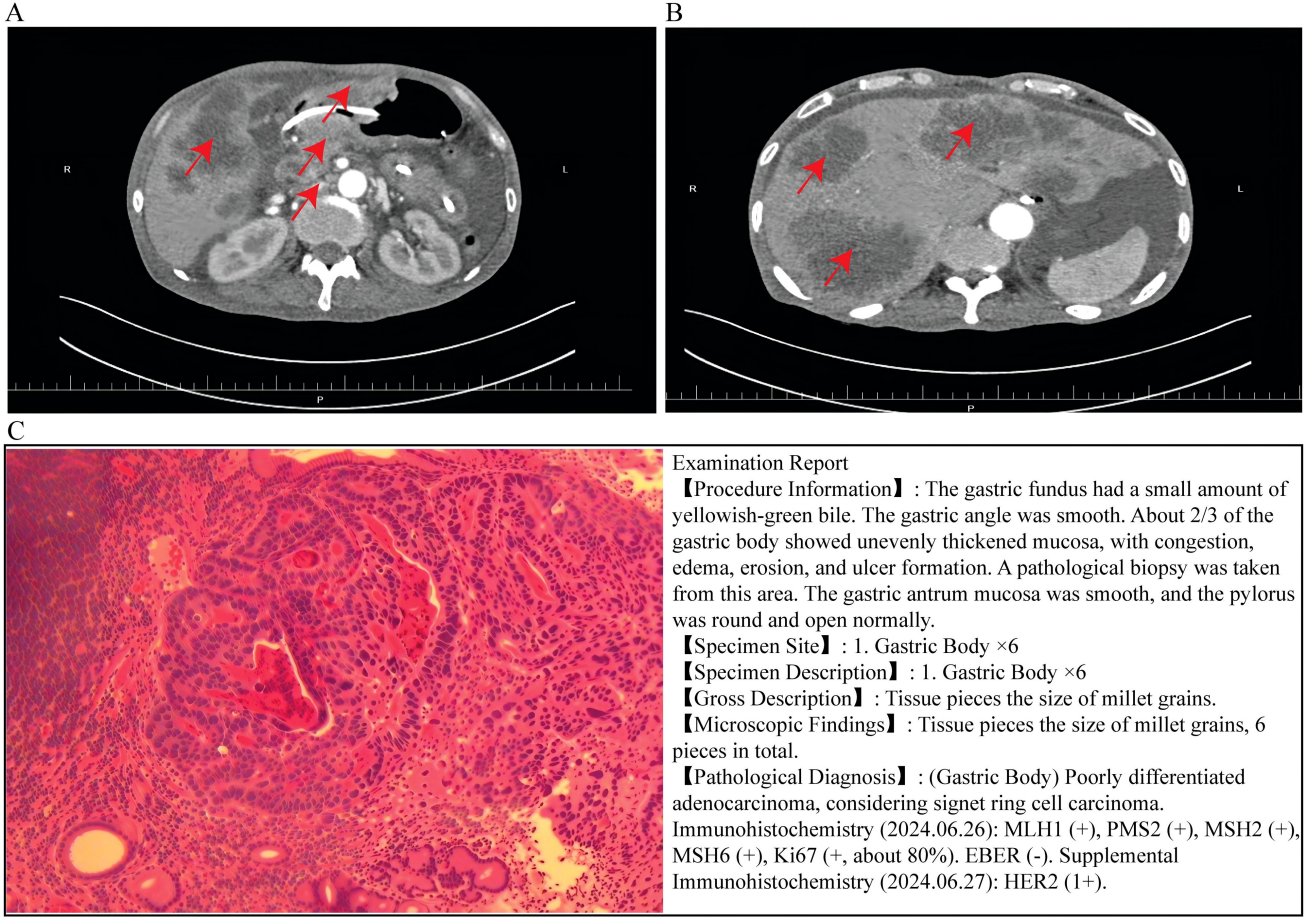
Imaging and pathological findings of patient. **(A)** Abdominal enhanced CT scan (axial view) showing multiple abnormal lesions (red arrows) in the abdominal cavity, suggesting possible local invasion or metastatic lesions. **(B)** Abdominal CT scan (axial view) revealing uneven thickening of the gastric body mucosa (red arrows), which is consistent with the endoscopic finding of gastric wall abnormality. **(C)** Hematoxylin-eosin (HE) staining of the gastric body biopsy specimen showing the morphological features of poorly differentiated adenocarcinoma (signet ring cell carcinoma component). The right panel displays the corresponding pathological examination report, confirming the diagnosis of (Gastric Body) poorly differentiated adenocarcinoma (considering signet ring cell carcinoma) and the results of immunohistochemical staining (MLH1 (+), PMS2 (+), MSH2 (+), MSH6 (+), Ki67 (+, ~80%), EBER (–), HER2 (1+)).

A 60-year-old man was admitted on September 5, 2024, with metastatic gastric cancer and a rapidly progressive generalized rash. He had been diagnosed with gastric adenocarcinoma with multiple hepatic metastases in January 2024. In June 2024, contrast-enhanced abdominal CT and gastroscopy confirmed a poorly differentiated adenocarcinoma of the gastric antrum with extensive nodal and liver metastases. Immunohistochemical staining confirmed MLH1(+), PMS2(+), MSH2(+), MSH6(+), Ki-67 (~80%), EBER (–), HER-2(1+), PD-L1 TPS <1%, and CPS ≈10, indicating a proficient mismatch repair (pMMR) status (**Figure 1**).

Given PD-L1 CPS ≥5, capecitabine plus oxaliplatin (XELOX) plus sintilimab was recommended; however, the family declined chemotherapy. The patient received sintilimab monotherapy (200 mg intravenously every 3 weeks) on August 21, 2024. Seven days later, he developed diffuse erythema and fever. Despite short-course methylprednisolone (60 mg/day for 3 days), the eruption rapidly worsened with extensive blistering and epidermal detachment, accompanied by hematemesis and melena. He received intravenous immunoglobulin (IVIG)(10 g) at an outside hospital but deteriorated and was transferred for further care. There was no prior history of drug allergy or newly introduced medications aside from sintilimab.

On arrival, vital signs were notable for hypotension (85/45 mmHg), tachycardia (102 bpm), and low-grade fever (37.5 °C). Dermatologic examination showed diffuse exfoliative dermatitis with bullae and sheet-like epidermal detachment involving >90% of body surface area. Residual necrotic epidermis and extensive erosions with copious exudation were present on the trunk and limbs. The severity-of-illness score for toxic epidermal necrolysis (SCORTEN) was 4, calculated using the worst values recorded during the first 48 h after admission, indicating high predicted mortality (13). Although the heart rate on admission was 102 bpm, the maximum heart rate during the first 48 hours reached 138 bpm, which contributed to the SCORTEN calculation. Occult blood in vomitus was positive. Laboratory tests demonstrated electrolyte disturbances, hypoalbuminemia (19 g/L), elevated lactate dehydrogenase (>1000 U/L),high-sensitivity c-reactive protein (70.86mg/L), aspartate aminotransferase (67.4U/L), Alanine Aminotransferase (11.5U/L), blood urea nitrogen (6.89mmol/L), creatinine (51.4umol/L), blood glucose (6.17mmol/L), blood bicarbonate levels (29.4mmol/L).

The diagnosis of sintilimab-induced toxic epidermal necrolysis (TEN) with concomitant upper gastrointestinal bleeding was made based on the temporal relationship (onset 7 days after the first sintilimab infusion), the characteristic clinical morphology (rapidly progressive diffuse erythema, bullae formation, and sheet-like epidermal detachment involving >90% of the body surface area), systemic manifestations (fever and hemodynamic instability), and exclusion of other potential triggers. Differential diagnoses included common drug eruption, autoimmune blistering diseases, and infectious exanthems. However, the extensive epidermal detachment and fulminant progression were not consistent with a typical morbilliform drug eruption, while autoimmune blistering diseases usually present with a more subacute course and different lesion patterns. Infectious rashes rarely cause widespread epidermal necrolysis. Given the absence of newly introduced medications other than sintilimab, sintilimab was considered the most likely causative agent and was permanently discontinued. Regarding the concurrent hematemesis and melena, the bleeding was clinically attributed to the primary gastric malignancy; however, as the extreme risk of severe mucosal injury and perforation during the acute phase of TEN precluded invasive endoscopy, the lack of acute-phase endoscopic confirmation remains a diagnostic limitation.

The patient was managed in an isolation unit. Aggressive fluid resuscitation and strict input/output monitoring were implemented. Hemostatic and acid-suppressive measures were administered for gastrointestinal bleeding. High-dose IVIG (20 g/day) was initiated, followed by carefully titrated systemic methylprednisolone once bleeding stabilized. Wound care followed burn management principles, including sterile saline irrigation, drainage of bullae with preservation of blister roofs when feasible, debridement of necrotic epidermis, topical recombinant human basic fibroblast growth factor (rh-bFGF) to promote re-epithelialization, and nanocrystalline silver dressings for exudative areas.

Wound cultures grew Escherichia coli, and antibiotic therapy was tailored accordingly based on antibiotic susceptibility testing. Specifically, the patient received intravenous piperacillin/tazobactam sodium (4.5 g every 8 hours). Because of severe catabolism and poor oral intake, enteral nutrition via jejunal feeding tube and parenteral nutrition were provided, along with albumin supplementation.

**Figure 2:**
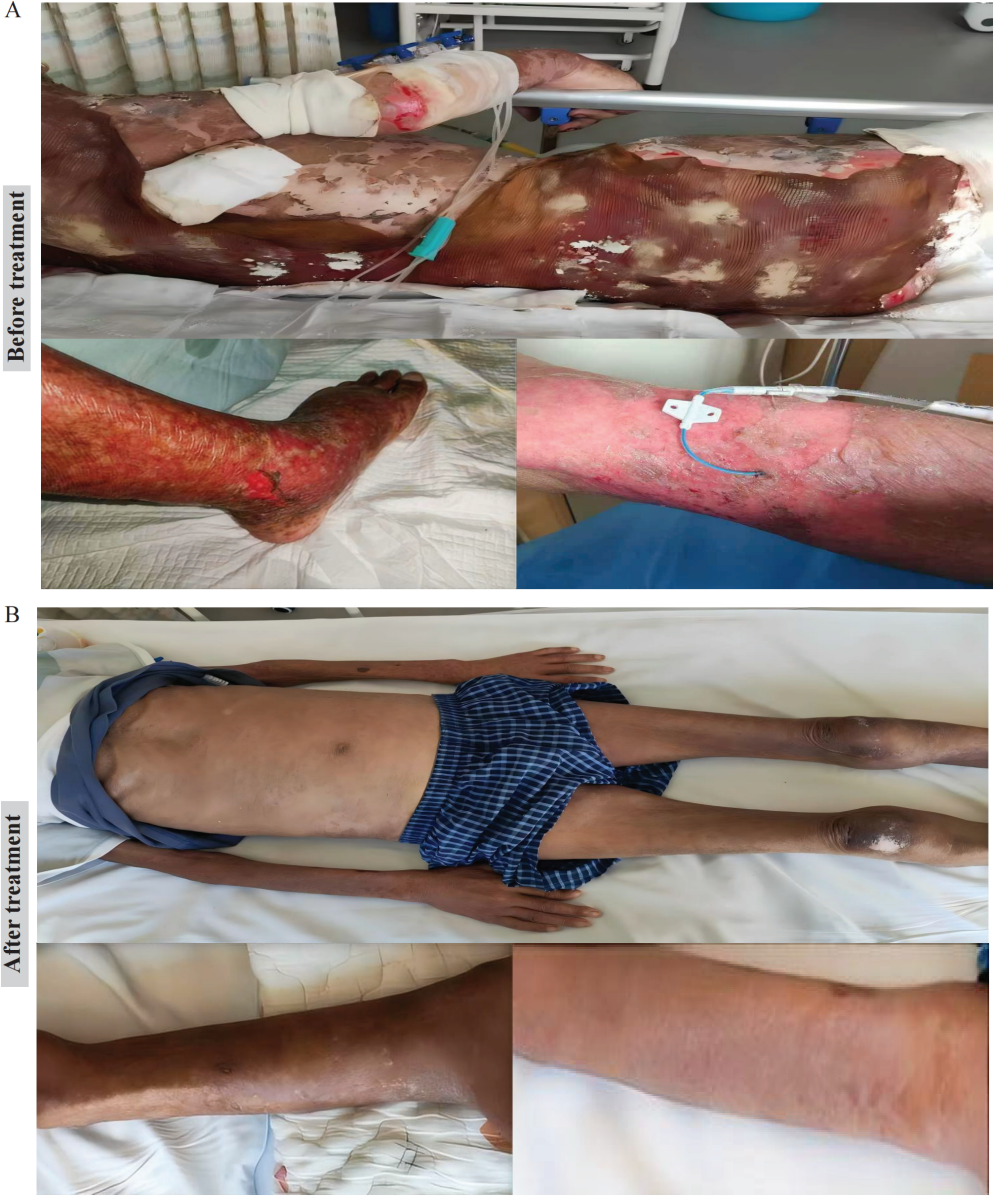
Comparison of skin lesions before and after treatment in this case of sintilimab-induced toxic epidermal necrolysis. **(A)** Before treatment/on admission, diffuse erythema with extensive bullae and widespread epidermal detachment involving >90% of the body surface area; prominent erosions, exudation, and necrotic epidermal remnants are evident on the trunk and extremities, consistent with severe TEN. **(B)** After discontinuation of the PD-1 inhibitor and multidisciplinary management including intravenous immunoglobulin, systemic corticosteroids, anti-infective therapy, wound care, and nutritional support, the lesions markedly improved with substantial re-epithelialization, reduced exudation, and residual mild hyperpigmentation/superficial scarring.

After 7 days of treatment, re-epithelialization became evident on the scalp and abdomen. By day 18, most limb lesions had healed; the back and buttocks improved more slowly due to pressure-related delay. By day 25, nearly all wounds had healed with only superficial scarring. The patient was discharged on October 7, 2024 (hospital day 32) with stable vital signs and no active bleeding (**Figure 2**). Follow-up showed no recurrence or secondary infection.

## 3 Discussion

This case demonstrates that even under extraordinarily complex circumstances – an advanced gastric cancer patient who developed *sintilimab-induced bullous epidermal necrolysis* complicated by gastrointestinal hemorrhage – timely and coordinated interventions can lead to successful control of the irAE and patient recovery. The patient’s severe SJS/TEN was effectively halted and reversed through early recognition, prompt discontinuation of immunotherapy, and a concerted multidisciplinary treatment strategy. This case adds to the limited existing literature on PD-1 inhibitor–associated TEN in gastric cancer, particularly in the setting of concurrent multi-organ complications The key clinical takeaway is that early, aggressive management of irAEs, even in the face of concurrent critical illness, can be life-saving. In our case, high-dose systemic corticosteroids were initiated as soon as BEN was suspected, despite concerns about steroid use in a patient with active bleeding. Supportive care measures – wound care akin to burn management, fluid/electrolyte management, infection prophylaxis, nutritional support – were instituted in parallel, under the guidance of an intensive care and burn-unit team. The gastrointestinal bleed was addressed concurrently via appropriate transfusions and interventions, allowing the patient to stabilize enough to heal. Ultimately, the patient recovered from BEN and resumed a nutritional and functional status close to baseline, highlighting that even *grade 4 irAEs* can be overcome with the right approach. This successful outcome reaffirms our hypothesis that multidisciplinary collaboration and decisive intervention were critical in averting mortality.

The development of SJS/TEN in the context of PD-1 blockade likely represents a convergence of immune dysregulation and host susceptibility. Under normal physiological conditions, PD-1 signaling restrains excessive T-cell activity; its inhibition can unleash potent cytotoxic responses that, in rare cases, become misdirected against host tissues. Mechanistic studies suggest that ICI-induced SJS/TEN is T-cell–mediated, similar to classic drug-induced cases, but with distinct immunologic dynamics.

Consistent with prior observations, PD-1 inhibitor–related SJS/TEN tends to occur after a delayed latency of several months (6), indicating a progressive breakdown of immune tolerance rather than an acute hypersensitivity reaction. One prevailing hypothesis proposes that PD-1 blockade lowers the activation threshold for autoreactive CD8^+^ cytotoxic T lymphocytes in the skin, while a secondary trigger—such as a concomitant medication or viral infection—precipitates widespread keratinocyte apoptosis.

In our patient, however, no new drug aside from sintilimab was identified before the onset of the rash, making sintilimab the most likely culprit. It is conceivable that the patient’s immune status (advanced malignancy, prior chemotherapy) and the inflammatory stress of tumor progression contributed to vulnerability. Notably, gastric cancer patients often have malnutrition and microbiome changes that could affect immunity; however, such factors are speculative in SJS/TEN risk. Genetic predispositions (e.g. HLA alleles linked to SJS in other drugs) remain undefined for checkpoint inhibitors, though a recent study did observe that non-White patients had nearly twice the risk of developing SJS/TEN from PD-1/PD-L1 therapy compared to White patients (6). Our patient, being Asian, fits this pattern.

## 4 Comparison with literature

Immune checkpoint inhibitor–associated SJS/TEN remains exceedingly rare, and current evidence is largely derived from isolated case reports and small case series. To date, two detailed cases of PD-1 inhibitor–induced SJS/TEN in patients with gastric cancer have been reported, while an additional gastric cancer case has been briefly mentioned in a recent literature review without detailed clinical description. Zhang et al. (14) described a case of sintilimab-induced TEN occurring 10 days after the first treatment cycle, which was refractory to systemic corticosteroids and intravenous immunoglobulin but showed rapid resolution after administration of adalimumab. Saada et al. (10) reported a case of nivolumab-induced SJS/TEN in metastatic gastric adenocarcinoma, which responded to high-dose corticosteroids and intensive supportive care. A recent comprehensive literature review of sintilimab-associated cutaneous adverse reactions identified 28 reported cases, among which lung cancer was the most common underlying malignancy, followed by gastrointestinal and other solid tumors. Notably, only a single patient with gastric cancer complicated by toxic epidermal necrolysis was mentioned in that review; however, this case was included solely as part of a summary table without detailed clinical description, treatment course, or outcome analysis (8). In addition to severe cutaneous toxicities, PD-1 inhibitors have also been reported to induce non-cutaneous, organ-specific immune-related adverse events in gastric cancer patients, such as nivolumab-associated Sjögren’s syndrome, further highlighting the broad spectrum of immune dysregulation triggered by these agents (15).

Consistent with these reports, our patient developed a sudden-onset, widespread blistering eruption with mucosal involvement following PD-1 inhibitor therapy, supporting the notion that severe cutaneous immune-related adverse events can occur early during treatment. However, our case demonstrates several clinically relevant distinctions. First, the clinical course was complicated by hemorrhagic shock secondary to acute gastrointestinal bleeding, a life-threatening condition rarely documented in previously reported ICI-associated SJS/TEN cases. Although the bleeding was most likely tumor-related rather than immune-mediated, it necessitated simultaneous management of two critical conditions and posed a substantial therapeutic dilemma regarding the timing and intensity of immunosuppression.

Second, compared with previously reported cases in gastric cancer, which predominantly required intensive care unit admission and prolonged hospitalization, our patient achieved stabilization under carefully balanced immunosuppressive therapy while managing concurrent systemic complications. This underscores the importance of early recognition of extensive blistering eruptions as a potential marker of systemic immune activation, even when other catastrophic events coexist. Notably, recent evidence suggests that PD-1 inhibitor–associated TEN may not represent an isolated cutaneous event, but rather part of a broader and evolving immune-mediated dermatologic spectrum. Zhang et al. (16). reported a patient with gastric adenocarcinoma who developed lichenoid dermatitis following recovery from sintilimab-induced TEN, supporting the concept of sequential or overlapping immune-related skin phenotypes after PD-1 blockade.

Finally, while the precise pathological mechanisms of PD-1 inhibitor–induced TEN remain incompletely understood, this case further supports the hypothesis that excessive activation of cytotoxic T-cell responses may extend beyond the skin and amplify vulnerability to systemic deterioration. Taken together, this case adds incremental value by illustrating the complex clinical decision-making required when severe cutaneous irAEs coexist with non-immune, life-threatening oncologic complications, thereby expanding the current understanding of risk stratification and management in this rare but fatal condition.

## 5 Innovations in management

Recent literature has explored adjunctive or alternative therapies for SJS/TEN, including TNF-α inhibitors (etanercept) and IV immunoglobulin (IVIG), both targeting inflammatory pathways implicated in keratinocyte apoptosis (9). Cyclosporine, which inhibits T-cell activation, has also shown promise in select reports (17).

In our patient, clinical improvement was achieved with corticosteroids alone, and thus second-line agents were not required. This outcome reinforces that high-dose systemic corticosteroids (1–2 mg/kg/day of IV methylprednisolone) remain the cornerstone for immune-mediated SJS/TEN, consistent with oncology guidelines for grade 4 skin irAEs (18). Although corticosteroid use in classic TEN is controversial due to infection risk, in ICI-induced cases, timely immunosuppression is generally essential to control hyperactive immune responses (19). Given the extensive skin loss, our patient also received broad-spectrum antibiotic prophylaxis to prevent sepsis, consistent with established TEN care protocols.

## 6 Clinical significance and implications

### 6.1 Early recognition

Early recognition of SJS/TEN is crucial to improving outcomes. The patient’s initial rash might have been mistaken for a benign eruption; prompt dermatologic consultation and skin biopsy confirmed the diagnosis. As emphasized in previous reports, any rash with mucosal involvement in an ICI-treated patient should be presumed to represent SJS/TEN until proven otherwise, and warrants immediate ICI discontinuation and initiation of high-dose corticosteroids.

Clinically, the rapid progression from diffuse erythema to blistering and extensive epidermal detachment within a short period after PD-1 inhibitor exposure should be regarded as a critical warning sign. Although this feature is not a validated predictor due to the rarity of ICI-associated TEN, it may help clinicians differentiate catastrophic bullous epidermal necrolysis from common immune-related rashes and trigger urgent dermatologic consultation. In our case, management required balancing immunosuppression with concurrent upper gastrointestinal bleeding. Hemodynamic stabilization and hemostatic measures were initiated immediately, while systemic corticosteroids were introduced after bleeding stabilization under close surveillance. This workflow may serve as a practical emergency framework; however, standardized protocols require validation in larger case series or registries.

### 6.2 Multidisciplinary management

The successful recovery in this case was achieved through multidisciplinary collaboration, echoing the recommendations in SJS/TEN guidelines (20). We convened a team comprising dermatology, oncology, intensive care, burn specialists, gastroenterology, ophthalmology, and nutrition. Each team played a vital role: dermatologists guided wound care and topical therapy; intensivists maintained hemodynamic stability and infection control; gastroenterologists managed bleeding; and nutritionists supported the patient’s hypercatabolic state. This approach aligns with SJS/TEN management guidelines, advocating care in specialized burn or ICU units under coordinated oversight. In complex irAEs, siloed care is suboptimal – only a coordinated approach will address the full scope of the syndrome.

### 6.3 Immunotherapy discontinuation and cancer control

Life-threatening irAEs such as TEN require permanent discontinuation of the offending ICI. Sintilimab was therefore stopped permanently in this case, and the patient was transitioned to palliative options. Rechallenge with PD-1 inhibitors is contraindicated following SJS/TEN due to high recurrence risk. This underscores the urgent need for predictive biomarkers—such as HLA genotyping or immune profiling—to identify high-risk patients before initiating immunotherapy.

### 6.4 Improving monitoring and early intervention

This case also highlights the need for proactive monitoring and patient education. The rash had progressed significantly before presentation, suggesting an opportunity for earlier intervention. Oncology clinics should emphasize prompt reporting of any skin changes and establish automatic dermatology consultation protocols for immunotherapy patients. Early steroid administration at grade 2 rash severity may prevent escalation to life-threatening reactions.

## 7 Limitations

As a single-case report, this study’s conclusions are inherently limited and cannot be generalized. While the close temporal relationship strongly implicates sintilimab as the likely trigger, definitive causality cannot be proven, and unrecognized contributing factors cannot be fully excluded. Mechanistic analyses—such as cytokine profiling, immunophenotyping, or T-cell clonality testing—were not performed, which could have provided deeper insight into the underlying immune activation pathways.

The optimal treatment strategy for ICI-induced SJS/TEN remains uncertain. While systemic corticosteroids were effective in this case, the potential roles of intravenous immunoglobulin, cyclosporine, or TNF-α blockade require validation in larger case series or prospective studies. Treatment choices in this case were highly individualized, as gastrointestinal bleeding and coagulopathy contraindicated procedures such as plasmapheresis. Though this pragmatic approach led to a favorable outcome, its applicability to other patients with differing clinical contexts remains to be determined.

## 8 Future directions

This case highlights the need for multicenter registries and collaborative research efforts to improve understanding of the pathogenesis, risk factors, and management of immune checkpoint inhibitor–induced SJS/TEN. Aggregated data may help identify shared clinical features and potential predictive markers, such as HLA alleles or cytokine signatures, and enable comparative evaluation of treatment strategies across different clinical settings.

From a clinical perspective, further discussion is warranted regarding the development of coordinated multidisciplinary approaches for managing grade 4 immune-related adverse events, integrating expertise from dermatology, oncology, intensive care, and related specialties. Such frameworks may facilitate earlier recognition and more timely intervention when severe toxicities occur.

Continued investigation into the immunogenetic mechanisms underlying these rare events is also needed. A better understanding of why only a subset of patients develop extreme immune-mediated reactions may eventually support risk stratification and preventive strategies. As immune checkpoint inhibitors are increasingly used in earlier-stage and adjuvant treatment settings, strengthening preparedness for rare but severe immune-related adverse events remains an important clinical objective.

## 9 Conclusions

Sintilimab can rarely induce life-threatening TEN in patients with advanced gastric cancer. Early recognition, immediate PD-1 inhibitor withdrawal, and rapid multidisciplinary intervention—including immunosuppression and burn-style supportive care—are essential for survival. This case highlights the clinical challenges posed by severe immune-related cutaneous adverse events occurring in the context of complex oncologic comorbidities, such as acute gastrointestinal bleeding. Although the favorable outcome observed in this patient underscores the potential effectiveness of prompt and coordinated management, conclusions regarding optimal treatment strategies should be interpreted with caution given the inherent limitations of a single-case report. Further accumulation of systematically documented cases and collaborative research efforts are needed to better define risk factors, pathophysiological mechanisms, and evidence-based management approaches for PD-1 inhibitor–associated SJS/TEN.

## Data Availability

The original contributions presented in the study are included in the article/supplementary material. Further inquiries can be directed to the corresponding authors.
